# High concentration and high dose of disinfectants and antibiotics used during the COVID-19 pandemic threaten human health

**DOI:** 10.1186/s12302-021-00456-4

**Published:** 2021-01-29

**Authors:** Zhongli Chen, Jinsong Guo, Yanxue Jiang, Ying Shao

**Affiliations:** Key Laboratory of the Three Gorges Reservoir Eco-Environment, Chongqing University, Chongqing, 400045 People’s Republic of China

## Abstract

The issue of coronavirus disease 2019 (COVID-19), caused by severe acute respiratory syndrome coronavirus-2 (SARS-CoV-2), has created enormous threat to global health. In an effort to contain the spread of COVID-19, a huge amount of disinfectants and antibiotics have been utilized on public health. Accordingly, the concentration of disinfectants and antibiotics is increasing rapidly in various environments, including wastewater, surface waters, soils and sediments. The aims of this study were to analyze the potential ecological environment impacts of disinfectants and antibiotics by summarizing their utilization, environmental occurrence, distribution and toxicity. The paper highlights the promoting effects of disinfectants and antibiotics on antibiotic resistance genes (ARGs) and even antibiotic resistant bacteria (ARB). The scientific evidences indicate that the high concentration and high dose of disinfectants and antibiotics promote the evolution toward antimicrobial resistance through horizontal gene transformation and vertical gene transformation, which threaten human health. Further concerns should be focused more on the enrichment, bioaccumulation and biomagnification of disinfectants, antibiotics, antibiotic resistance genes (ARGs) and even antibiotic resistant bacteria (ARB) in human bodies.

## Introduction

As the coronavirus disease 2019 (COVID-19) epidemic worsens, various disinfectants and pharmaceuticals have been actively used to control the spread of the virus. Disinfectants, such as alcohols, chlorine, formaldehyde, glutaraldehyde, ortho-phthalaldehyde, hydrogen peroxide, iodophors, peracetic acid, phenolics, and quaternary ammonium compounds were recommended to use alone or in combinations on public health by authorities. The Chinese Center for Disease Control and Prevention (China CDC), United States Environmental Protection Agency (US EPA) and World Health Organization (WHO) successively released the guidance for disinfectant utilization to against the virus, while the lack of necessary training program for public could raise the adverse effects of disinfectant on human health [1]. Besides disinfectants, mass production and considerable pharmaceuticals, in particular antibiotics, are applied to treat bacterial infections. For instance, in the United States alone, over 250 million antibiotic prescriptions are written annually. In China, 96 million kg antibiotics were used in 2007. These antibiotics cannot be completely metabolized or eliminated in the body, and 30–90% of which are excreted unchanged into the waste system [2]. Traditional wastewater treatment processes can only remove 20–80% of pharmaceuticals and their metabolites [3]. Therefore, either directly or indirectly, these disinfectants and antibiotics will eventually be emitted in environments. Accordingly, disinfectants and antibiotics were frequently detected in surface waters, ground waters, soils, sediments and wetlands, with the amounts up to 1 mg/L, the environmental impacts therefore become a worldwide concern [4].

**Fig. 1:**
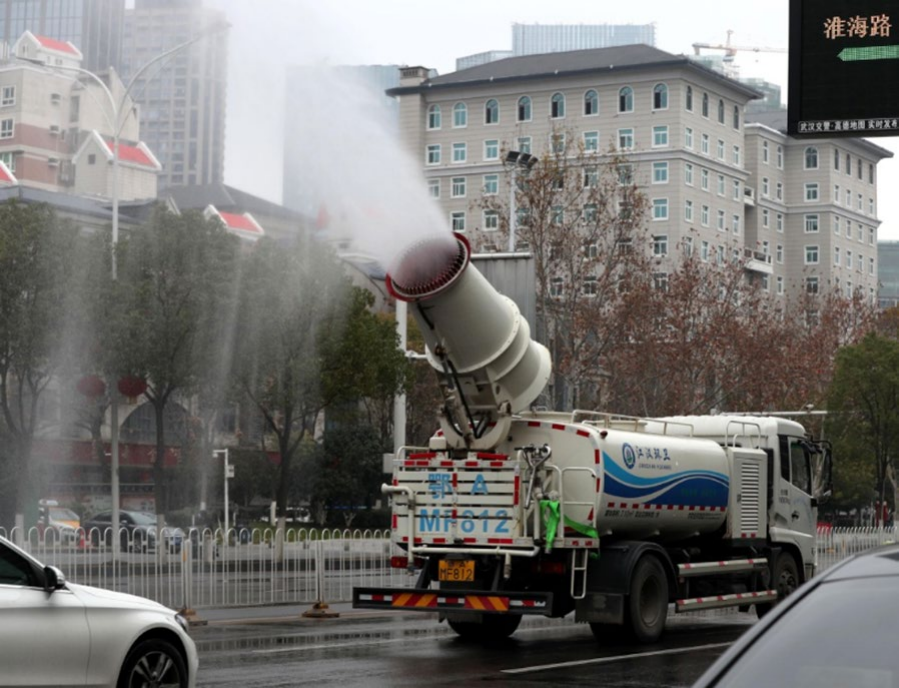
Disinfection and sterilization in Wuhan, China. The figure was cited from the China Daily website on April 13, 2020

In the times of COVID-19 panic, disinfectants and antibiotics were overused for the coronavirus disease control and treatment, since the effective treatment measures and medications are not yet determined. Until 2020 March, China has dispensed at least 2000 tons of disinfectants in the city of Wuhan alone (Fig. 1) [5]. Even though the novel coronavirus is virus, and antibiotics cannot been used for treatment the disease of COVID-19 directly, numerous antibiotics were widely utilized to resist the COVID-19-induced inflammation and other disease. These increasing use and misuse will definitely increase the concentration and dose of disinfectants and antibiotics in environments. Thus, can the high concentration and high dose of disinfectants and antibiotics during the COVID-19 pandemic lead to more stress on ecological safety and human health?

## Toxicity

Considering that disinfectants and antibiotics are designed for the treatment of bacterial infections, it is reasonable to assume that non-target environmental organism and/or microorganism are more likely to be affected due to non-therapeutic exposure [6]. Many environmental studies hence focus on the occurrence of disinfectants by-products (DBPs) and antibiotics residuals in diverse environments and their toxic effects on various organisms [7–9]. DBPs and antibiotics in surface waters and sewerage treatment plant effluents/wastewaters are generally measured at concentrations ranging between 0.01 and 1.0 μg/L [10]. Many DBPs were proved to inhibit the growth of freshwater algae and duckweeds by disturbing the function of photosynthesis through inhibition and interference of chloroplast metabolism at environmental concentrations [10]. Most of the emerging DBPs were found to induce oxidative stress, DNA damage, and activate DNA repair system at environmental concentrations [11]. Chronic toxicological studies pointed that exposure to DBPs may induce genotoxicity, cytotoxicity, asthma, skin rashes, bladder and colon cancer in humans [12]. The frequently detected DBPs of trichloromethane, dibromochloromethane, bromodichloromethane, tribromomethane, dichloroacetonitrile and dibromoacetonitrile have hence recommended specific maximum values at 200, 100, 60, 100, 20, and 70 μg/L, respectively, in the WHO Guidelines [13]. Antibiotics can affect prokaryotic cells by inhibition of cell envelope synthesis, protein synthesis and nucleic acid (DNA/RNA) synthesis [10]. Environmental exposures of antibiotics, such as diclofenac, erythromycin, fluoxetine and carbamazepine, caused gill alterations, renal lesions and oxidative DNA damage in rainbow trout, delay in Zebrafish hatching, growth inhibition in cyanobacteria and green algae, neurotoxicity and behavioral changes [14]. It is noted that that the more frequent home use of disinfectants was associated with dysbiosis in infant gut microbiota, which may lead to childhood overweight and obesity [15]. Therefore, the environmental risk assessment is required for a medicine if the predicted environmental concentration exceeds 10 ng/L in Europe [16].

## Antimicrobial resistance evolution

Disinfectant by-products and antibiotic residues permanently existed in diverse environments, which can persistently promote bacterial evolution toward antimicrobial resistance (AMR). Bacteria, only carrying antibiotic resistance genes (ARGs), can survive and persist in these contaminated environments. A disinfection by-products study reported that disinfection by-products can select strain with high-level resistance via broadly conserved cellular functions and pathways at the minimal inhibitory concentrations [17].

In fact, ARGs and antibiotic resistant bacteria (ARB) can be target selected in disinfectants and antibiotics contaminated environments.[18]. The ARGs spread among bacteria, namely horizontal gene transformation (HGT), which induced the enrichment effects of ARGs in ARB through the uptake of naked DNA and mobile genetic elements such as plasmids, transposons, integrons, gene cassettes, and bacteriophages [17, 19]. Expansions in the diversity and abundance of ARGs were hence presented in water, soil and air, which may disturb the normal microflora. Emergence of resistance amongst bacteria in the normal flora and distribution of resistant genes can contribute to an increased load of resistant, potentially pathogenic microorganisms and reduce the colonization resistance leading to overgrowth of exogenic pathogens [20]. New ARB, such as the COVID-19, could be a result of ARGs enrichment and microflora disturbance, and it hence increased and spread rapidly all over the world in recent years. Conversely, the huge amounts of ARGs and ARB would promote the evolution of microbial structure through ecological niches occupation, which will enhance the pressure of target selection. Consequently, it may induce the imbalance of microbial ecosystem.

Besides HGT, ARGs and ARB can also be transferred from environments or microorganisms to higher trophic levels through drinking water, food and respiration [21]. Human health studies reported that the ARGs and ARB were frequently detected in animal and human gut. A dietary exposure study reported that the number and abundance of ARGs significantly increased in the collembolan gut microbiome after antibiotic exposure for 14 days [22]. Many clinically relevant ARGs are found to originate from non-pathogenic environmental bacteria. These ARGs and ARB lead to the reduction of the effectiveness of antimicrobial drugs during clinical practice, and finally will implicate human health. It is estimated that AMR causes 700,000 deaths annually worldwide. If no proper action is taken, these number could grow to 10 million per year by 2050 [23]. The WHO hence issued in 2017 that AMR has become a critical global public health issue of this century.

## Conclusion

COVID-19 has threatened the global health remarkably. High concentration and high dose of disinfectants and antibiotics used during the COVID-19 pandemic, which entered into environments, are definitely accelerating the target selection of AMR in environments [24]. HGT among bacteria increases the enrichment of ARGs in ARB. Vertical gene transformation through food chain put the trophic level organisms in a high risk of AMR bioaccumulation and biomagnification. Human beings, at the top of the food chain, are actually exposed in a mixture of high level of disinfectants, antibiotics, ARGs and ARB. Environment studies focusing only on the damage of chemicals or AMR with ignoring the mixture effects is not enough. Further researches have to pay more attention on the enrichment, bioaccumulation and biomagnification of disinfectants, antibiotics, ARGs and even ARB in human bodies. The rapid detection technologies for COVID-19 have to be accelerated development, which could provide useful information on guiding the usage of disinfectants and antibiotics [25]. Management on solid and liquid waste is required to update for adaptation the changes of lifestyle during the COVID-19 outbreak, including developing flexible waste treatment plans and the corresponding training, and establishment longer-term systematic assessments [26, 27]. Ecological techniques such as constructed wetlands which can remove disinfectants and antibiotics by sunlight photo degradation, biodegradation and adsorption should be applied for ameliorating the impacts of disinfectants and antibiotics in aquatic environments [28].

## Data Availability

The datasets used and/or analyzed during the current study are available from the corresponding author on reasonable request.

## Abbreviations

COVID-19: Coronavirus disease 2019
SARS-CoV-2: Severe acute respiratory syndrome coronavirus-2
CDC: The Chinese Center for Disease Control and Prevention
EPA: United States Environmental Protection Agency
WHO: World Health Organization
DBPs: Disinfectants by-products
AMR: Antimicrobial resistance
ARGs: Antibiotic resistance genes
ARB: Antibiotic resistant bacteria
HGT: Horizontal gene transformation
